# *In Vitro* Ability of Currently Available Oximes to Reactivate Organophosphate Pesticide-Inhibited Human Acetylcholinesterase and Butyrylcholinesterase

**DOI:** 10.3390/ijms12032077

**Published:** 2011-03-23

**Authors:** Daniel Jun, Lucie Musilova, Kamil Musilek, Kamil Kuca

**Affiliations:** 1 Center of Advanced Studies, Faculty of Military Health Sciences, University of Defence, Trebesska 1575, Hradec Kralove, 500 01, Czech Republic; 2 Department of Water Resources and Environmental Modeling, Faculty of Environmental Sciences, Czech University of Life Sciences Prague, Kamycka 129, Praha 6—Suchdol, 16521, Czech Republic; 3 University Hospital Hradec Kralove, Sokolska 581, Hradec Kralove, 50005, Czech Republic; 4 Hospital Pharmacy, University Hospital Hradec Kralove, Sokolska 581, Hradec Kralove, 500 05, Czech Republic; E-Mail: musilova.lucie@fnhk.cz; 5 Department of Biochemical Sciences, Charles University in Prague, Faculty of Pharmacy in Hradec Kralove, Heyrovskeho 1203, Hradec Kralove, 50005, Czech Republic; 6 Department of Toxicology, Faculty of Military Health Sciences, University of Defence, Trebesska 1575, Hradec Kralove, 50001, Czech Republic; E-Mail: musilek@pmfhk.cz

**Keywords:** acetylcholinesterase, butyrylcholinesterase, *in vitro*, nerve agent, organophosphate, pesticide, reactivator, oxime, scavenger

## Abstract

We have *in vitro* tested the ability of common, commercially available, cholinesterase reactivators (pralidoxime, obidoxime, methoxime, trimedoxime and HI-6) to reactivate human acetylcholinesterase (AChE), inhibited by five structurally different organophosphate pesticides and inhibitors (paraoxon, dichlorvos, DFP, leptophos-oxon and methamidophos). We also tested reactivation of human butyrylcholinesterase (BChE) with the aim of finding a potent oxime, suitable to serve as a “pseudocatalytic” bioscavenger in combination with this enzyme. Such a combination could allow an increase of prophylactic and therapeutic efficacy of the administered enzyme. According to our results, the best broad-spectrum AChE reactivators were trimedoxime and obidoxime in the case of paraoxon, leptophos-oxon, and methamidophos-inhibited AChE. Methamidophos and leptophos-oxon were quite easily reactivatable by all tested reactivators. In the case of methamidophos-inhibited AChE, the lower oxime concentration (10^−5^ M) had higher reactivation ability than the 10^−4^ M concentration. Therefore, we evaluated the reactivation ability of obidoxime in a concentration range of 10^−3^–10^−7^ M. The reactivation of methamidophos-inhibited AChE with different obidoxime concentrations resulted in a bell shaped curve with maximum reactivation at 10^−5^ M. In the case of BChE, no reactivator exceeded 15% reactivation ability and therefore none of the oximes can be recommended as a candidate for “pseudocatalytic” bioscavengers with BChE.

## 1. Introduction

Organophosphates (OPs) are widely used as pesticides (e.g., parathion, methamidophos) in agriculture, as plasticizers and flame retardants (cresyl diphenyl phosphate, triaryl phosphate) in the industry, and as toxic chemical warfare agents (nerve agents, e.g., sarin, VX). OP pesticide poisonings causes tens of thousands of deaths every year in the whole world [1]. OP pesticides irreversibly inhibit enzymes acetylcholinesterase (AChE; EC 3.1.1.7) and butyrylcholinesterase (BChE; EC 3.1.1.8) by phosphorylation or phosphonylation (phosphylation) of the serine hydroxyl group at the enzyme’s active site. Inhibited AChE cannot cleave the neuromediator acetylcholine which is then accumulated in the nerve synapses and causes cholinergic crisis, which can lead to death [2]. Resulting enzyme complexes underlie secondary reactions, which may affect the efficacy of medical treatment and the diagnosis [3]. Phosphylated AChE and BChE may undergo spontaneous dealkylation, through alkyl–oxygen bond scission or P–N bond scission in case of tabun [3–5]. This unimolecular process is called aging and it results in an irreversibly inactivated enzyme [3]. Negatively charged adduct is then stabilized by interaction with the catalytic His 440 [6]. Reactivation is limited by aging of the AChE and high concentrations of pesticides. Aging of AChE takes longer with diethyl-OPs compounds than with dimethyl-OPs; half-lives of dimethyl-OPs are 3.7 h, and of diethyl-OPs, 31 h. For this reason, the oxime therapy should begin as soon as possible following intoxication. Oximes may be effective if started within about 120 hours for diethyl-OP poisoning and 12 hours for dimethyl-OP poisoning (therapeutic window, four times the half-life) [7–10]. Additional secondary reaction is a spontaneous reactivation, leading to recovery of AChE or BChE function [3]. Spontaneous reactivation is the combination of two parallel processes: dephosphylation and aging [6]. Kinetics of aging, reactivation and spontaneous dephosphylation depends on the source of cholinesterase and chemical structure of OP and reactivator.

Current therapy of OP poisonings involves the administration of anticholinergic drug (atropine), anticonvulsants (diazepam) and for the recovery of function of inhibited AChE antidotes from the group of pyridinium or bispyridinium aldoximes (oximes) are used. Therapeutic efficacy of these compounds depends on their chemical structure and also type of OP inhibitor. Prophylaxis is realized by administration of reversible inhibitors of AChE (e.g., carbamate pyridostigmine) [11]. Relatively new approach in treatment of OP poisonings is using of enzyme bioscavengers. These bioscavengers are able to catch and neutralize toxic OP molecules in the bloodstream, before they can reach their physiological target—AChE in different tissues [12]. They can prevent post-exposure incapacitation and toxic effects that are commonly observed in animals treated by traditional antidotal regimen [13]. Many scientific efforts have been focused on butyrylcholinesterase (BChE) as a bioscavenger capable of sequestering molecules of nerve agents or pesticides from the bloodstream and serving as OP-nonspecific prophylactics [12,14]. BChE was recently evaluated in several clinical studies (phase I) in USA.

**Figure 1.**
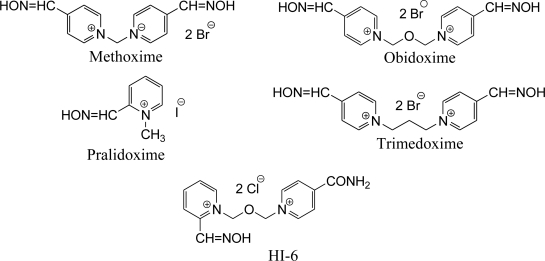
Structures of tested oxime reactivators.

The aim of our work was to test and summarize reactivation ability of common, commercially available AChE reactivators (methoxime, pralidoxime, obidoxime, trimedoxime and HI-6, see Figure 1) and to evaluate their potential usefulness in the treatment of OP pesticide poisoning.

We have tested their ability to reactivate human AChE inhibited by five structurally different pesticides *in vitro*. Moreover, we tested reactivation of BChE, in parallel, with the aim of finding a potent oxime, suitable of serving in combination with this enzyme (administered as prophylactic antidote or occurring naturally in blood) as a “pseudocatalytic” bioscavenger. Such a combination could allow an increase of prophylactic and therapeutic efficacy of the administered enzyme and also a decrease in the amount of enzyme necessary and cost of such a bioscavenger [15,16].

## 2. Results and Discussion

Measured values of reactivation ability of tested oxime reactivators are summarized in Tables 1 and Table 2.

Reactivators were evaluated *in vitro*, in concentrations 10^−4^ M and 10^−5^ M, which are usually attainable in plasma within clinical treatment in the hospital in the case of intoxication by OPs [20].

**Figure 2.**
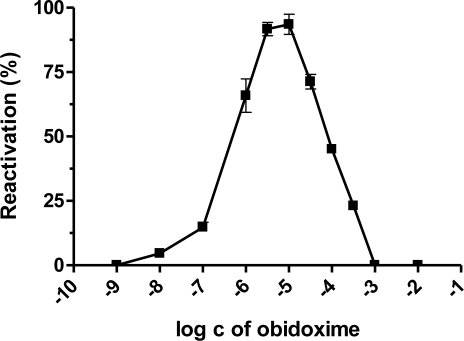
Relationship between obidoxime concentration and corresponding reactivation ability of methamidophos-inhibited AChE.

**Figure 3.**
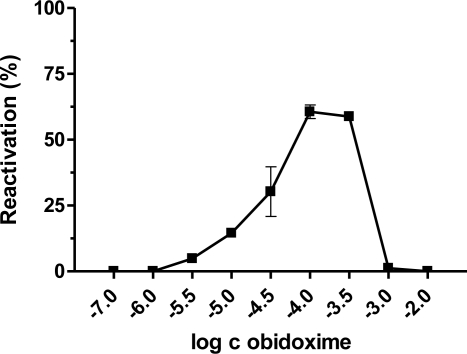
Relationship between obidoxime concentration and corresponding reactivation ability of paraoxon-inhibited AChE.

**Figure 4.**
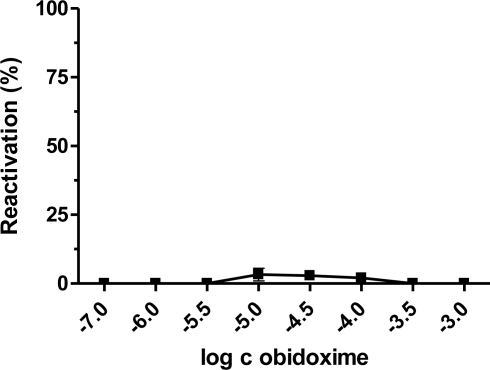
Relationship between obidoxime concentration and corresponding reactivation ability of dichlorvos-inhibited AChE.

**Figure 5.**
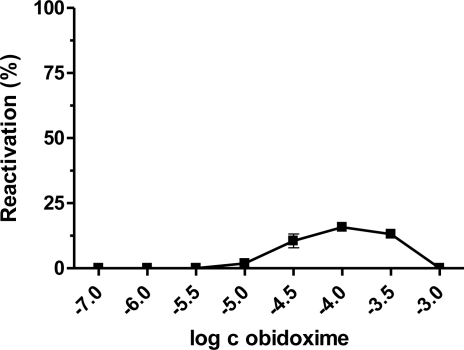
Relationship between obidoxime concentration and corresponding reactivation ability of DFP-inhibited AChE.

**Figure 6.**
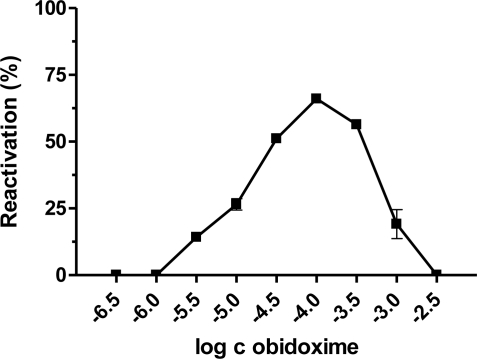
Relationship between obidoxime concentration and corresponding reactivation ability of leptophos-oxon-inhibited AChE.

Our results demonstrated that the best broad-spectrum AChE reactivators after 10 minutes of reactivation, are trimedoxime and obidoxime, because they reached more than 50% of reactivation in the case of paraoxon, leptophos-oxon and methamidophos-inhibited AChE. DFP- and dichlorvos-inhibited AChE were not sufficiently reactivated. Methamidophos and leptophos-oxon are quite easily reactivatable by all tested reactivators. In reactivation of dichlorvos-, paraoxon-, DFP- and leptophos-oxon-inhibited AChE, a higher oxime concentration caused higher reactivation of the phosphorylated enzyme. Reverse behavior was observed for methamidophos-inhibited enzyme, where the maximum reactivation ability was achieved with lower oxime concentration. Therefore, we investigated the relationship between oxime concentration and type of inhibitor used. Obidoxime was selected as a suitable model oxime and its reactivation ability was estimated in a concentration range (depending on the inhibitor) of 10^−8^–10^−2^ M (Figures 2–6). As a result, we obtained bell-shaped dependencies of reactivation ability on obidoxime concentration for each inhibitor.

Maximum level of reactivation for each inhibitor was achieved at different obidoxime concentrations and, especially for methamidophos; the maximum was shifted towards lower concentrations. While the lower oxime concentration (left side of the graph), causes practically only reactivation of the phosphorylated enzyme, on increasing concentration enzyme inhibition with reactivator occurs (right side of the graph) [21]. This is the reason, why the course of the reactivation curve is always bell-shaped in the whole concentration range of oxime [22,23]. Therefore, the reactivation process itself is characterized in the increasing and the decreasing parts of the curve showing both reactivation and inhibition of liberated intact AChE by the reactivator itself. Each reactivator varies in the optimal concentration for reactivation. Lower concentrations were optimal for some oximes (e.g., 10^−5^ M) [22,24,25]. Another important factor affecting the reactivation process and the course of the reactivation curve is the formation of phosphorylated oximes during reactivation [26] as their presence causes re-inhibition of the enzyme. The probability of their formation increases with higher enzyme or oxime concentrations [27]. There is evidence that phosphorylated oximes could decompose in blood because human plasma paraoxonase may be capable of degrading dimethoxy- and diethoxy-phosphorylated oximes derived from obidoxime and trimedoxime [28].

Aging of inhibited enzyme could also decrease reactivation potency. In this study, measured reactivation ability was not corrected for this phenomenon.

According to our results, reactivation ability of tested oximes for BChE was very low in comparison with values measured for AChE. Comparable results, with reactivation of OP-inhibited BChE, were also obtained by Aurbek *et al.* [29].

## 3. Experimental Section

Cholinesterase reactivators used in this study were synthesized in our lab or purchased from Leciva (Czech Republic), Merck (Germany) and Phoenix Chemicals Ltd. (United Kingdom). Purity of all the AChE reactivators utilized was tested using TLC (DC-Alufolien Cellulose F; mobile phase *n*-butanol-acetic acid-water = 5:1:2; detection by Dragendorff reagent) and NMR (Varian Gemini 300, Palo Alto, CA, USA), and their purity was higher than 98%. Pesticides and inhibitors with a minimum purity of 90% were purchased from Dr. Ehrenstorfer (Augsburg, Germany) and Sigma-Aldrich (Czech Republic). All other chemicals used in this study were of analytical purity and were purchased from Sigma-Aldrich (Czech Republic). Reactivation potency of five oximes (methoxime, pralidoxime, obidoxime, trimedoxime and HI-6) was tested by *in vitro* screening test. For this purpose modified Ellman’s method was used [17–19,25,30–32]. Paraoxon (diethyl 4-nitrophenyl phosphate, the active “toxic” form of pesticide parathion), dichlorvos (2,2-dichlorovinyl dimethyl phosphate), DFP (diisopropyl fluorophosphate), leptophos-oxon (*O*-[4-bromo-2,5-dichlorophenyl] *O*-methyl phenylphosphonate) and methamidophos (*O*,*S*-dimethyl phosphoramidothioate) were selected as suitable model OP inhibitors. Human erythrocyte hemolyzate was used as a source of AChE and human plasma as a source of BChE. The blood samples were collected from healthy volunteers from the vein into a disposable syringe containing 3.8% sodium citrate (the ratio blood/citrate was 1:10 *w*/*w*). The citrated blood was centrifuged for 20 min at 2856 × g, the plasma was removed as supernatant, frozen and was kept under −80 °C (source of BChE). The erythrocytes were washed three times with phosphate buffer (PB; 0.1 M, pH 7.4) and then hemolyzed in PB (0.01 M, pH 7.4) in a ratio 1:10 (*w*/*w*), frozen and kept under −80 °C (source of AChE). Enzymes were inhibited to 5 % of their original activity using concentration corresponding to IC_95_ (concentration which causes 95% inhibition). Time of enzyme inhibition (equivalent to 7 × T_1/2_) was calculated separately for each compound from experimentally determined half-life (T_1/2_) of reaction between enzyme and inhibitor. Detailed data about used concentrations of inhibitors and time of inhibition are summarized in Table 3. The inhibition of AChE was started in plastic cuvettes by addition of inhibitor solution in isopropanol to the mixture of phosphate buffer (0.05 M, pH 7.4) and hemolyzate (activity before inhibition was set to 10 U/L) or plasma (activity was set to 13.3 U/L). Concentration of isopropanol in the sample was 5%. This concentration had no significant influence on the activity of both cholinesterases. Blind samples with uninhibited enzyme were incubated for given time (Table 3) with isopropanol in final concentration 5% [25,32]. Inhibited enzymes were then immediately incubated for 10 min with solution of reactivator in phosphate buffer (0.05 M, pH 7.4) at concentrations 10^−4^ M and 10^−5^ M (and 10^−8^–10^−2^ M for obidoxime). Mixture was diluted 20 times before the measurement. Activity of AChE (BChE) was determined spectrophotometrically by modified method according to Ellman [30]. The final concentration of DTNB and acetylthiocholine or butyrylthiocholine in the mixture was 10^−3^ M.

All results were corrected for oximolysis. Reactivation potency was calculated from the formula:

$$\%\;R=[1-(a_{0}-a_{r})/(a_{0}-a_{i})]\times 100$$

where % *R* is percent of reactivation, *a_0_* is activity of intact enzyme, *a_i_* is activity of inhibited enzyme and *a_r_* is activity of reactivated enzyme minus oximolysis. Data were not corrected for aging because enzyme was reactivated at the latest one hour after inhibition and time of incubation with oxime was relatively short (10 min.) Detailed description of the method can be found in publications of Musilova *et al.* [31,32].

## 4. Conclusions

According to our results, bisquaternary oximes seem to be more potent reactivators of pesticide-inhibited AChE than monoquaternary pralidoxime. No reactivator achieved sufficient ability to reactivate OP pesticide-inhibited BChE, and therefore none of the oximes can be recommended as a candidate for “pseudocatalytic” bioscavengers with BChE. Because of this, a larger group (not only the currently available ones) of reactivators need to be tested to better understand the structure–activity relationship which would then help in the synthesis of new reactivators of BChE—“rational synthesis of BChE reactivators”.

## Figures and Tables

**Table 1.** Reactivation ability of five oximes to reactivate OP-inhibited human erythrocyte AChE, at concentrations 10^−4^ M and 10^−5^ M (%, mean value of three independent determinations; time of reactivation 10 min; pH 7.4; temperature 25 °C).

AChE	Paraoxon [17]				Dichlorvos				DFP [18]				Leptophos-oxon [19]				Methamidophos			

	100 μM		10 μM		100 μM		10 μM		100 μM		10 μM		100 μM		10 μM		100 μM		10 μM	

	Mean	SD	Mean	SD	Mean	SD	Mean	SD	Mean	SD	Mean	SD	Mean	SD	Mean	SD	Mean	SD	Mean	SD
Methoxime	23.4	4.5	4.3	1.5	0	0	0.2	0.6	6.4	0.5	1.5	0.5	52.6	0.5	11.9	0.9	61.7	2.4	68.1	11.4
Pralidoxime	18.1	0.9	1.3	0.9	2.6	0.6	0.2	0.6	2.3	0.2	0	0	13.2	0.9	4.1	1.3	53.4	3.1	53.8	22.6
Obidoxime	96.8	0.9	59.4	0.9	0	0	0.6	0.1	17.1	0.1	7.4	0.5	50.3	0.9	31.4	0.2	5.8	4.8	57.0	18.7
Trimedoxime	86.0	1.6	45.3	0.8	0	0	0	0	23.8	0.2	6.4	0.2	51.3	0.5	26.4	2.7	9.4	7.5	53.1	10.9
HI-6	16.1	0.2	3.9	0.9	0	0	0.6	1.1	0	0	0	0	32.8	8.0	11.6	0.4	37.4	12.3	75.2	14.6

**Table 2.** Reactivation ability of five oximes to reactivate OP-inhibited human plasma BChE, at concentrations 10^−4^ M and 10^−5^ M (%, mean value of three independent determinations; time of reactivation 10 min; pH 7.4; temperature 25 °C).

BChE	Paraoxon [17]				Dichlorvos				DFP [18]				Leptophos-oxon [19]				Methamidophos			

	100 μM		10 μM		100 μM		10 μM		100 μM		10 μM		100 μM		10 μM		100 μM		10 μM	

	Mean	SD	Mean	SD	Mean	SD	Mean	SD	Mean	SD	Mean	SD	Mean	SD	Mean	SD	Mean	SD	Mean	SD
Methoxime	6.1	0.6	0.9	0.8	0.2	0.1	0.2	0.1	8.2	1.3	0.8	0.5	6.4	0.4	1.9	1.8	4.8	0.2	1.0	0.2
Pralidoxime	5.5	0.1	1.0	0.3	1.0	0.1	0.4	0.2	6.4	0.8	0.7	0.1	2.3	1.8	0	0	3.5	0.3	0	0
Obidoxime	9.9	0.4	2.2	0.4	3.1	0.2	1.6	0.4	9.5	1.0	1.5	0.6	14.3	0.6	6.5	4.2	4.2	0.3	1.0	0.2
Trimedoxime	12.1	1.7	1.3	0.3	1.2	0.1	0.4	0.2	7.3	0.5	0.8	0.5	8.5	2.4	2.1	0.4	5.2	0.7	0.6	0.8
HI-6	2.3	0.3	0.8	0.5	0.6	0.1	0.4	0.1	3.8	0.1	0.7	0.2	5.6	4.9	0	0	4.8	0.2	0.1	0.2

**Table 3.** Concentration of inhibitors (IC_95_) and time of inhibition (7 × T_1/2_) for AChE and BChE used in experiment.

**Inhibitor**	**AChE**		**BChE**	

	**IC_95_ (M)**	**7 × T_1/2_ (min)**	**IC_95_ (M)**	**7 × T_1/2_ (min)**
Paraoxon	3.38 × 10^−6^	2.17	1.41 × 10^−7^	1.82
Dichlorvos	3.30 × 10^−4^	0.32	2.08 × 10^−6^	2.20
DFP	5.00 × 10^−6^	0.93	8.30 × 10^−8^	1.75
Leptophos*−*oxon	4.16 × 10^−7^	2.45	7.06 × 10^−6^	0.75
Methamidophos	4.26 × 10^−5^	2.22	2.08 × 10^−4^	2.83

## References

[1] Costa LG (2006). Current issues in organophosphate toxicology. Clin. Chim. Acta.

[2] Maxwell DM, Brecht KM, Koplovitz I, Sweeney RE (2006). Acetylcholinesterase inhibition: Does it explain the toxicity of organophosphorus compounds?. Arch. Toxicol.

[3] Worek F, Koller M, Thiermann H, Szinicz L (2005). Diagnostic aspects of organophosphate poisoning. Toxicology.

[4] Elhanany E, Ordentlich A, Dgany O, Kaplan D, Segall Y, Barak R, Velan B, Shafferman A (2001). Resolving pathways of interaction of covalent inhibitors with the active site of acetylcholinesterases: MALDI-TOF/MS analysis of various nerve agent phosphyl adducts. Chem. Res. Toxicol.

[5] Barak D, Ordentlich A, Kaplan D, Barak R, Mizrahi D, Kronman C, Segall Y, Velan B, Shafferman A (2000). Evidence for P-N bond scission in phosphoroamidate nerve agent adducts of human acetylcholinesterase. Biochemistry.

[6] Millard CB, Kryger G, Ordentlich A, Greenblatt HM, Harel M, Raves ML, Segall Y, Barak D, Shafferman A, Silman I, Sussman JL (1999). Crystal structures of aged phosphonylated acetylcholinesterase: Nerve agent reaction products at the atomic level. Biochemistry.

[7] Eyer P (2003). The role of oximes in the management of organophosphorus pesticide poisoning. Toxicol. Rev.

[8] Worek F, Backer M, Thiermann H, Szinicz L, Mast U, Klimmek R, Eyer P (1997). Reappraisal of indications and limitations of oxime therapy in organophosphate poisoning. Hum. Exp. Toxicol.

[9] Thompson DF, Thompson GD, Greenwood RB, Trammel HL (1987). Therapeutic dosing of pralidoxime chloride. Drug Intell. Clin. Pharm.

[10] Eddleston M, Singh S, Buckley N (2003). Acute organophosphorus poisoning. Clin Evid.

[11] Bajgar J, Fusek J, Kuca K, Bartosova L, Jun D (2007). Treatment of organophosphate intoxication using cholinesterase reactivators: Facts and fiction. Mini. Rev. Med. Chem.

[12] Saxena A, Sun W, Luo C, Myers TM, Koplovitz I, Lenz DE, Doctor BP (2006). Bioscavenger for protection from toxicity of organophosphorus compounds. J. Mol. Neurosci.

[13] Maxwell DM, Brecht KM, Doctor BP, Wolfe AD (1993). Comparison of antidote protection against soman by pyridostigmine, HI-6 and acetylcholinesterase. J. Pharmacol. Exp. Ther.

[14] Kolarich D, Weber A, Pabst M, Stadlmann J, Teschner W, Ehrlich H, Schwarz HP, Altmann F (2008). Glycoproteomic characterization of butyrylcholinesterase from human plasma. Proteomics.

[15] Lenz DE, Yeung D, Smith JR, Sweeney RE, Lumley LA, Cerasoli DM (2007). Stoichiometric and catalytic scavengers as protection against nerve agent toxicity: A mini review. Toxicology.

[16] Jun D, Musilova L, Link M, Loiodice M, Nachon F, Rochu D, Renault F, Masson P (2010). Preparation and characterization of methoxy polyethylene glycol-conjugated phosphotriesterase as a potential catalytic bioscavenger against organophosphate poisoning. Chem. Biol.Inter.

[17] Jun D, Musilova L, Kuca K, Kassa J, Bajgar J (2008). Potency of several oximes to reactivate human acetylcholinesterase and butyrylcholinesterase inhibited by paraoxon *in vitro*. Chem. Biol. Inter.

[18] Bajgar EJ (2009). Central and Peripheral Nervous System: Effects of Highly Toxic Organophosphates and Their Antidotes.

[19] Jun D, Musilova L, Pohanka M, Jung YS, Bostik P, Kuca K (2010). Reactivation of human acetylcholinesterase and butyrylcholinesterase inhibited by leptophos-oxon with different oxime reactivators *in vitro*. Int. J.Mol. Sci.

[20] Bajgar J (2004). Organophosphates/nerve agent poisoning: Mechanism of action, diagnosis, prophylaxis, and treatment. Adv. Clin. Chem.

[21] Lorke DE, Nurulain SM, Hasan MY, Kuca K, Musilek K, Petroianu GA (2008). Eight new bispyridinium oximes in comparison with the conventional oximes pralidoxime and obidoxime: *In vivo* efficacy to protect from diisopropylfluorophosphate toxicity. J. Appl. Toxicol.

[22] Musilek K, Kuca K, Jun D, Dohnal V, Dolezal M (2005). Synthesis of a novel series of bispyridinium compounds bearing a xylene linker and evaluation of their reactivation activity against chlorpyrifos-inhibited acetylcholinesterase. J. Enzyme Inhib. Med. Chem.

[23] Kuca K, Cabal J, Jun D, Hrabinova M (2006). In vitro evaluation of acetylcholinesterase reactivators as potential antidotes against tabun nerve agent poisonings. Drug Chem. Toxicol.

[24] Kuca K, Kassa J (2003). A comparison of the ability of a new bispyridinium oxime-1-(4-hydroxyiminomethylpyridinium)-4-(4-carbamoylpyridinium)butane dibromide and currently used oximes to reactivate nerve agent-inhibited rat brain acetylcholinesterase by *in vitro* methods. J. Enzyme Inhib. Med. Chem.

[25] Kuca K, Musilova L, Palecek J, Cirkva V, Paar M, Musilek K, Hrabinova M, Pohanka M, Karasova JZ, Jun D (2009). Novel bisquaternary oximes—Reactivation of acetylcholinesterase and butyrylcholinesterase inhibited by paraoxon. Molecules.

[26] Stenzel J, Worek F, Eyer P (2007). Preparation and characterization of dialkylphosphoryl-obidoxime conjugates, potent anticholinesterase derivatives that are quickly hydrolyzed by human paraoxonase (PON1192Q). Biochem. Pharmacol.

[27] Worek F, Diepold C, Eyer P (1999). Dimethylphosphoryl-inhibited human cholinesterases: Inhibition, reactivation, and aging kinetics. Arch. Toxicol.

[28] Kiderlen D, Worek F, Klimmek R, Eyer P (2000). The phosphoryl oxime-destroying activity of human plasma. Arch. Toxicol.

[29] Aurbek N, Thiermann H, Eyer F, Eyer P, Worek F (2009). Suitability of human butyrylcholinesterase as therapeutic marker and pseudo catalytic scavenger in organophosphate poisoning: A kinetic analysis. Toxicology.

[30] Ellman GL, Courtney KD, Andres V, Feather-Stone RM (1961). A new and rapid colorimetric determination of acetylcholinesterase activity. Biochem. Pharmacol.

[31] Musilova L, Jun D, Palecek J, Cirkva V, Musilek K, Paar M, Hrabinova M, Pohanka M, Kuca K (2010). Novel nucleophilic compounds with oxime group as reactivators of paraoxon-inhibited cholinesterases. Lett. Drug Design Disc.

[32] Musilova L, Kuca K, Jung YS, Jun D (2009). *In vitro* oxime-assisted reactivation of paraoxon-inhibited human acetylcholinesterase and butyrylcholinesterase. Clin. Toxicol. (Phila).

